# Changes in Brain Lateralization in Patients with Mild Cognitive Impairment and Alzheimer’s Disease: A Resting-State Functional Magnetic Resonance Study from Alzheimer’s Disease Neuroimaging Initiative

**DOI:** 10.3389/fneur.2018.00003

**Published:** 2018-02-08

**Authors:** Hao Liu, Lele Zhang, Qian Xi, Xiaohu Zhao, Fei Wang, Xiangbin Wang, Weiwei Men, Qixiang Lin

**Affiliations:** ^1^Tongji University School of Medicine, Shanghai, China; ^2^Department of Imaging, Changping District Hospital, Beijing, China; ^3^Department of Radiology, Shanghai East Hospital, Tongji University School of Medicine, Shanghai, China; ^4^Department of Imaging, The Fifth People’s Hospital of Shanghai, Fudan University, Shanghai, China; ^5^Department of Imaging, Shanghai TongJi Hospital, Shanghai, China; ^6^Department of Neurosurgery, Shanghai TongJi Hospital, Shanghai, China; ^7^Institute of Heavy Ion Physics, Peking University, Beijing, China; ^8^Center for MRI Research, Beijing Key Laboratory for Medical Physics and Engineering, Beijing, China; ^9^National Key Laboratory of Cognitive Neuroscience and Learning, Beijing Normal University, Beijing, China

**Keywords:** resting-state functional magnetic resonance imaging, brain lateralization, intrinsic laterality index, mild cognitive impairment, Alzheimer’s disease

## Abstract

**Purpose:**

To detect changes in brain lateralization in patients with mild cognitive impairment (MCI) and Alzheimer’s disease (AD) using resting-state functional magnetic resonance imaging (fMRI).

**Materials and methods:**

Data from 61 well-matched right-handed subjects were obtained from the Alzheimer’s Disease Neuroimaging Initiative, including 19 healthy controls (HCs), 25 patients with MCI, and 17 patients with AD. First, we divided 256 pairs of seed regions from each hemisphere covering the entire cerebral gray matter. Then, we used the intrinsic laterality index (iLI) approach to quantify the functional laterality using fMRI. One-way ANOVA was employed to estimate the differences in iLI among the three groups. The sum, number and mean value of the iLI were calculated within the thresholds of 0 < |iLI| < 0.2, 0.2 ≤ |iLI| < 0.4, 0.4 ≤ |iLI| < 0.8, and |iLI| ≥ 0.8, to explore the changes in the lateralization of resting-state brain function in patients with MCI and AD.

**Results:**

One-way ANOVA revealed that the iLIs of the three groups were significantly different. The HCs showed a significant leftward interhemispheric difference within |iLI| ≥ 0.8. Compared with the HCs, the patients with MCI manifested a distinct abnormal rightward interhemispheric asymmetry, mainly within the thresholds of 0.2 ≤ |iLI| < 0.4 and 0.4 ≤ |iLI| < 0.8; in the patients with AD, the normal leftward lateralization that was observed in the HCs disappeared, and an abnormal rightward laterality was expressed within 0.4 ≤ |iLI| < 0.8. By directly comparing the patients with MCI with the patients with AD, an exclusive abnormal rightward laterality was observed in the patients with MCI within the 0.2 ≤ |iLI| < 0.4 threshold, and the normal leftward asymmetry vanished in the patients with AD within the |iLI| ≥ 0.8 threshold.

**Conclusion:**

Global brain lateralization was different among three groups. The abnormal rightward dominance observed in the patients with MCI and AD may indicate that these patients use additional brain resources to compensate for the loss of cognitive function, and the observed disappearance of the leftward laterality in the patients with AD was likely associated with the damage in the left hemisphere. The observed disappearance of the rightward asymmetry in the patients with AD using the 0.2 ≤ |iLI| < 0.4 threshold was likely a sign of decompensation. Our study provides new insights that may improve our understanding of MCI and AD.

## Introduction

The two brain hemispheres differ in anatomy and function in humans. Regarding brain laterality, the left hemisphere is more specialized in language processing (1, 2), while the right hemisphere is more specialized in attention, musical abilities, visuospatial tasks, and many aspects of emotion (3, 4). Regarding brain structural asymmetries, the left hemisphere has a larger planum temporale, the right hemisphere has a larger hippocampus and the lateral fissure in the left hemisphere is longer than that in the right hemisphere (5–7). In addition, the degree of the structural asymmetry was found to be correlated with the degree of functional lateralization in right-handed individuals (8). Biduła also revealed that there were significant associations between the anatomical asymmetries in the insular cortex and the lateralization of functional activity in both gesture planning and language (Pearson’s *r* = 0.6, *r* = 0.49) (9).

Brain asymmetry is believed to be evolutionarily adaptive. Functional specialization that primarily involves processing within one hemisphere may allow the expanded human brain to minimize between-hemisphere connectivity and distribute domain-specific processing functions (10). Furthermore, hemispheric specialization is hypothesized to contribute to rapid and efficient information processing (11, 12).

Hemispheric asymmetries have been shown to change in healthy aging as follows: when performing a cognitive task, the activity in the frontal lobes in healthy elderly subjects is less lateralized than that in young subjects (13, 14). In addition, brain injury or neurological disorders in which the anatomical and functional integrity of the brain are impaired, such as Alzheimer’s disease (AD) and schizophrenia, also result in alterations in brain asymmetry (15–18). Hippocampal asymmetry was found to be significantly reduced in subjects with dementia (15). Additionally, Long et al. showed that compared with healthy elderly subjects, age-matched groups of patients with mild cognitive impairment (MCI) and AD featured a greater progressive loss of structural asymmetry in the causal anterior cingulate cortex, parahippocampal gyrus, and entorhinal cortex (19). Although the cortical structural asymmetry pattern observed in healthy elderly subjects was generally maintained in the patients with MCI and AD, there was a progressive decrease in the degree of asymmetry (20). However, the changes in the cerebral asymmetry in the cognitively impaired patients are primarily in the structural domain. In this study, we applied a general method based on whole-brain analyses and functional magnetic resonance imaging (fMRI) to investigate the changes in brain lateralization in subjects with MCI and AD. To quantify brain lateralization, we used an intrinsic laterality index (iLI) (21) to investigate 256 × 256 paired regions of MCI and AD patients compared to healthy controls (HCs). Different from studies about structural asymmetry and certain types of functional asymmetry under tasks, iLI shows the brain lateralization of intrinsic functional connectivity at resting state. Our goal is to reach a more comprehensive understanding of the changes in hemispheric asymmetry and the differences in brain lateralization in patients with MCI and AD from that of healthy elderly subjects.

## Materials and Methods

### Overview of Alzheimer’s Disease Neuroimaging Initiative (ADNI)

The data used in this study were downloaded from the ADNI database.^1^ The ADNI was launched in 2003 by the National Institute on Aging (NIA), the National Institute of Biomedical Imaging and Bioengineering, the Food and Drug Administration, private pharmaceutical companies, and non-profit organizations. The primary goal of the ADNI was to determine whether the use of MRI, positron emission tomography, other biological markers, and clinical and neuropsychological assessments can be combined to measure the progression of MCI and early stages of AD. The identification of the characteristics of very early AD progression will be helpful for researchers and clinicians to develop new treatments, monitor their effects, and reduce the time and cost of clinical trials. For up-to-date information, see www.adni-info.org. The ADNI is an open database, researchers could download imaging and clinical data from the website, the institutional review board approval documents are not generally available.

### Participants

Sixty-four right-handed subjects participated in this study and were categorized into groups of HCs (*n* = 20), patients with MCI (*n* = 25), and patients with AD (*n* = 19). The HCs had Mini–Mental State Examination (MMSE) scores between 26 and 30 (inclusive) and Clinical Dementia Rating (CDR) scores of 0 or 0.5; the patients with MCI had MMSE scores between 23 and 30 (inclusive) and CDR scores of 0, 0.5, or 1; and the patients with AD had MMSE scores between 12 and 27 (inclusive) and CDR scores of 0.5, 1, or 2. The fMRI data, which correspond to structural MRI data, and the clinical data of all subjects were downloaded from the publicly available ADNI database prior to July 24, 2015. The data of three subjects (one HC and two patients with AD) were excluded due to excessive motion (see Data Preprocessing). Details regarding the clinical and demographic data of the remaining 61 subjects are shown in Table 1. There were no significant differences in gender or age among the groups.

**Table 1:** Demographics and clinical information.

Characteristics	Healthy control (*n* = 19)	Mild cognitive impairment (*n* = 25)	Alzheimer’s disease (*n* = 17)	*P*-value
Age	74.48 ± 6.56	74.25 ± 7.38	74.08 ± 8.09	0.528^a^
Female (%)	52.63	68.00	70.59	>0.900^b^
Mini–Mental State Examination	28.84 ± 1.01	27.96 ± 2.01	20.76 ± 3.53	<0.001^a^

*Data are presented as the means ± SD*.

*^a^The P-value was obtained using a one-way analysis of variance*.

*^b^The P-value was obtained using the Pearson chi-square test*.

### Data Acquisition

All subjects were scanned using a 3.0-T Philips MRI scanner. Resting-state functional images were obtained using an echo-planar imaging sequence with the following parameters: 140 time points, repetition time (TR) = 3,000 ms, echo time (TE) = 30 ms, flip angle = 80°, number of slices = 48, slice thickness = 3.3 mm, spatial resolution = 3 mm × 3 mm × 3 mm and matrix = 64 × 64. Whole-brain coverage included the entire cerebellum. Subjects were instructed to stay awake, keep their eyes open, and minimize head movement; no other task instructions were provided. The corresponding brain structural images were obtained using a 3D T1-weighted magnetization-prepared rapid gradient echo sequence with the following parameters: TR = 6.8 ms, TE = 3.1 ms, flip angle = 9°, slice thickness = 1.2 mm, number of slices = 170, sagittal images, spatial resolution = 1 mm × 1 mm × 1.2 mm and matrix = 256 × 256.

### Image Preprocessing

Data were preprocessed using the Data Processing Assistant for Resting-State fMRI^2^ (22), which is based on Statistical Parametric Mapping software (SPM8)^3^ and Resting-State fMRI Data Analysis Toolkit (REST^4^) (23). The resting-state data were preprocessed using the following steps: (1) the first five image volumes of resting-state data were discarded to ensure signal equilibrium and subjects’ adaptation to the fMRI scanning noise. (2) The remaining 135 images were corrected for the timing differences between each slice. (3) Rigid body corrections were implemented for head motion (six-parameter rigid body) and datasets with more than 1.5 mm of maximum displacement in any of the *x, y*, or *z* directions or 1.5° of any angular motion were discarded, resulting in the exclusion of three subjects (one HC and two patients with AD). (4) The realigned images were spatially normalized to the standard echo-planar imaging template, based on the Montreal Neurological Institute stereotactic space, and then resampled them into 3 mm × 3 mm × 3 mm cubic voxels. First, fMRI data for each subject were linearly registered with their own T1 structural images, then images were registered to the standard space using united segmentation, and registration parameters were written into fMRI data. (5) Data were spatially smoothed with a Gaussian kernel of 6 mm × 6 mm × 6 mm full width at half maximum (FWHW) to decrease spatial noise and (6) the linear trends and temporal filter (0.01 Hz < *f* < 0.08 Hz) were retained. Parameters for the white matter signal, global mean signal, and cerebrospinal fluid signal were used as nuisance variables in the functional connectivity analysis to remove any residual effects of motion and other non-neuronal factors. In the present study, we did not remove the image frames based on head motion because Zeng et al. indicated that the data scrubbing procedure might cause inflated connectivity estimates in specific regions (24).

### Definition of Seed Regions

**Figure 1:**
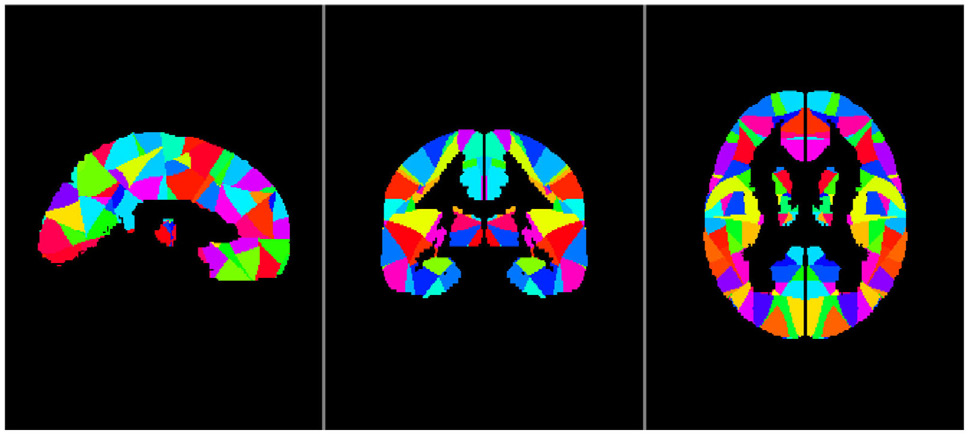
Sample images of seed regions, from left to right: sagittal image, coronal image, and axial image. The left and right hemisphere is completely symmetrical.

Our analyses were based on the whole brain; therefore, we divided the brain, including the entire cerebral gray matter and excluding the cerebral white matter and cerebellum, into 256 pairs of seed regions (25). Each subject had 512 seed regions. First, we removed all white matter and cerebellum from the MNI-ICBM152 Symmetric template, and then we randomly divided the gray matter in the left hemisphere into 256 seed regions; certain voxels were included in a region and not all the regions were the same size. The pattern of divided regions in the left hemisphere was generated with mirror symmetry in the right hemisphere. Three images are shown in Figure 1 as examples of seed regions.

### Functional Connectivity

To evaluate functional connectivity, mean preprocessed resting fMRI time course were extracted from volume of regions of interest in the cerebral gray matter. Pearson’s product–moment correlation was then computed between the time course of corresponding seed regions and target regions. Correlation coefficients were converted to *z* values using Fisher’s *r*-to-*z* transform to improve the normality (26).

### Calculation of the iLI

**Figure 2:**
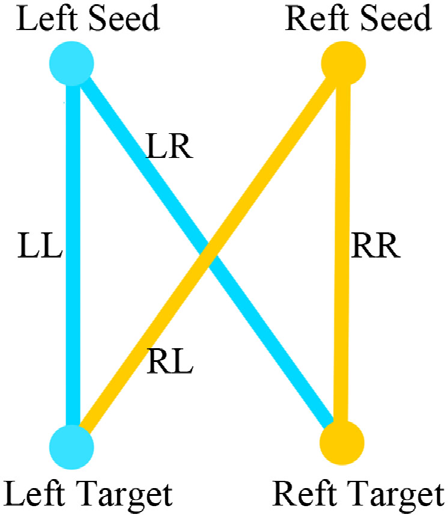
LL is the strength of the correlation between the left-hemisphere target region and the left-hemisphere seed region; LR represents the strength of the correlation between the left seed region and the right target region; and RR and RL represent the contralateral homologs (21).

We used iLI (21) to quantify brain functional connectivity lateralization. A total of 512 seed regions were defined. For each pair of seed regions within one hemisphere, the homologous regions in the opposite hemisphere were identified and used to derive a laterality index based on the relative functional correlation strengths among the four regions (see Figure 2). According to these four seed-target correlations, iLI was then calculated using the following equation:

$$\text{Laterality Index=}\frac{\left(\text{LL}-\text{RL}\right)-\left(\text{RR}-\text{LR}\right)}{\left|\text{LL}\right|\text{+}\left|\text{RL}\right|\text{+}\left|\text{RR}\right|\text{+}\left|\text{LR}\right|}.$$

**Figure 3:**
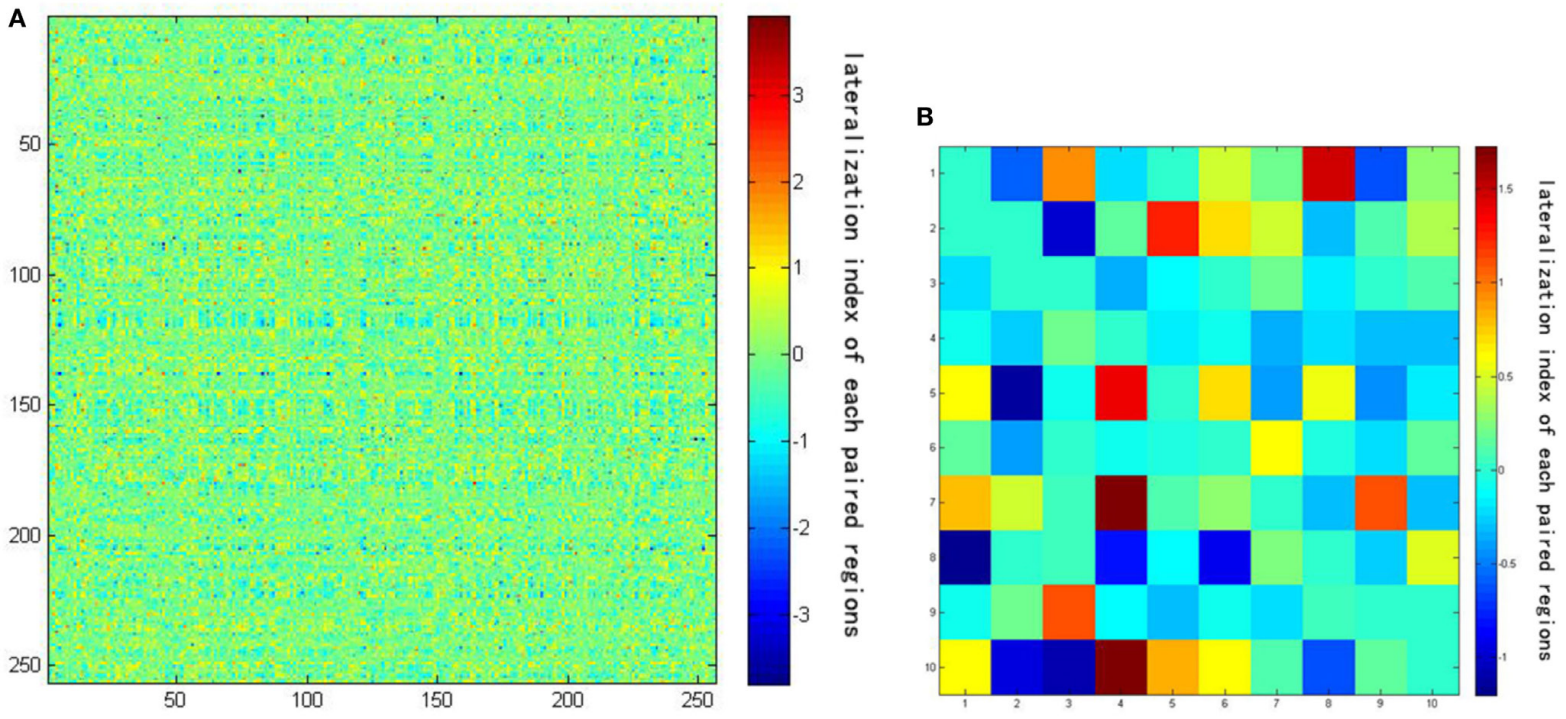
**(A)** The result of lateralization index of one subject using a 256 × 256 matrix. **(B)** The amplification of the first 10 × 10 matrix of the result. Darker colors represent lower intrinsic laterality index values.

LL is the strength of the correlation between the left-hemisphere target region and the left-hemisphere seed region; LR represents the strength of correlation between the left seed and the right target; and RR and RL represent the contralateral homologs. A positive value for iLI indicates left lateralization and a negative value indicates right lateralization. The higher the absolute value of iLI, the more lateralized the seed region. When the denominator was less than 0.2, iLI was set to 0. The iLI was computed for all 256 seed regions in each hemisphere against the 255 possible target regions; we also set the iLI of each pair of seed region against itself to 0, yielding 65,536 pairwise correlations for each subject. The results of each subject are shown in the form of a 256 × 256 matrix (see Figure 3).

### Statistical Analyses

One-way ANOVA was employed to estimate differences in the iLI among the groups to test whether whole-brain lateralization was different among the groups. Moreover, we computed the sums, numbers, and mean values for left-lateralized iLI and right-lateralized iLI, for each subject using the following four thresholds: 0 < |iLI| < 0.2, 0.2 ≤ |iLI| < 0.4, 0.4 ≤ |iLI| < 0.8, and 0.8 ≤ |iLI|, in order to test a “local” lateralization tendency. The threshold is somewhat arbitrary, but all lateralized iLIs were included and the threshold was selected to reduce the number of iLIs to an appropriate number and to detect whether changes in brain lateralization were observed between patients with MCI and the patients with AD. Statistical analyses were performed using the Statistical Package for the Social Science (SPSS 20.0). Paired-samples *t*-tests were used to assess the whole hemispheric differences in lateralization within groups. Corresponding sum, number or mean values for left-lateralized iLI and right-lateralized iLI were used as paired variables. A *P*-value < 0.05 was considered to indicate significant asymmetry.

Considering that sex differences may influence brain lateralization, we compared the iLI according to gender among the three groups. Independent two-samples *t*-tests were used to evaluate the iLIs of male and female subjects in each group.

## Results

**Figure 4:**
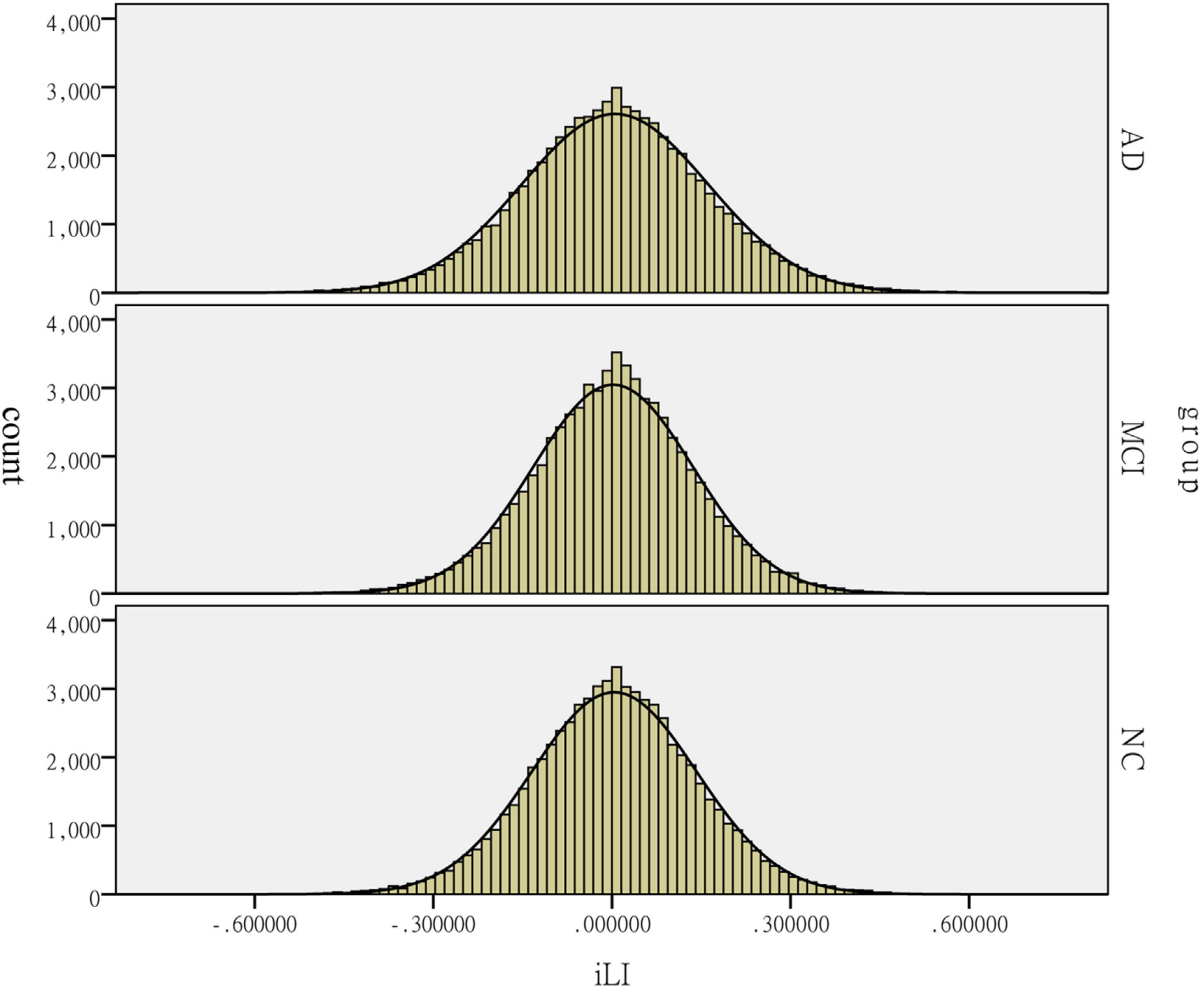
Distribution of the intrinsic laterality index (iLI) among the patients with Alzheimer’s disease (AD) and mild cognitive impairment (MCI) and the healthy controls (HCs). The histograms reflect the lateralization tendency among the groups. The horizontal axis presents the iLI values, whereas the vertical axis presents the count.

The one-way ANOVA of iLI revealed distinct differences among the groups (*P* = 0.001). The laterality distribution of the iLI among the three groups is illustrated in Figure 4. Based on the results of the within-group analysis, the pattern of brain lateralization changed in the patients with MCI and AD. The results of the paired-samples *t*-test are shown in Tables 2–4.

**Table 2:** Statistical analysis of the sum of the iLI at different threshold values using the paired-samples *t*-test.

	Groups	SLL	SRL	*P*-value
Healthy control	0 < |iLI| < 0.2	1,086.75 ± 73.40	1,096.07 ± 78.75	0.37
	0.2 ≤ |iLI| < 0.4	2,056.80 ± 100.06	2,096.47 ± 103.01	0.29
	0.4 ≤ |iLI| < 0.8	4,447.39 ± 356.08	4,603.84 ± 389.62	0.21
	0.8 ≤ |iLI|	5,544.76 ± 739.74	5,072.07 ± 785.54	0.005
Mild cognitive impairment	0 < |iLI| < 0.2,	1,083.83 ± 83.12	1,078.86 ± 72.59	0.38
	0.2 ≤ |iLI| < 0.4	2,084.79 ± 54.94	2,125.88 ± 76.99	0.03
	0.4 ≤ |iLI| < 0.8	4,527.50 ± 277.75	4,705.29 ± 414.88	0.01
	0.8 ≤ |iLI|	5,525.25 ± 772.06	4,855.01 ± 1,158.53	0.003
Alzheimer’s disease	0 < |iLI| < 0.2,	1,105.09 ± 100.86	1,122.61 ± 85.33	0.20
	0.2 ≤ |iLI| < 0.4	2,072.47 ± 89.19	2,123.25 ± 53.22	0.07
	0.4 ≤ |iLI| < 0.8	4,347.15 ± 349.36	4,546.64 ± 469.81	0.039
	0.8 ≤ |iLI|	5,401.92 ± 583.02	4,949.52 ± 712.94	0.06

*SLL, sum of the left-lateralized laterality index; SRL, sum of the right-lateralized laterality index; iLI, intrinsic laterality index*.

**Table 3:** Statistical analysis of the number of the iLI at different threshold values using the paired-samples *t*-test.

	Groups	NLL	NRL	*P*-value
Healthy control	0 < |iLI| < 0.2	11,678.16 ± 903.39	11,754.42 ± 954.22	0.39
	0.2 ≤ |iLI| < 0.4	7,061.21 ± 341.29	7,188.58 ± 346.46	0.32
	0.4 ≤ |iLI| < 0.8	7,688.63 ± 598.08	7,944.84 ± 658.91	0.23
	0.8 ≤ |iLI|	5,185.89 ± 653.86	4,824.95 ± 686.16	0.009
Mild cognitive impairment	0 < |iLI| < 0.2	11,573.36 ± 1,053.28	1,521.80 ± 951.17	0.24
	0.2 ≤ |iLI| < 0.4	7,146.44 ± 187.77	7,277.52 ± 245.80	0.04
	0.4 ≤ |iLI| < 0.8	7,833.32 ± 460.91	8,124.48 ± 693.02	0.01
	0.8 ≤ |iLI|	5,026.28 ± 485.32	4,680.88 ± 623.90	0.005
Alzheimer’s disease	0 < |iLI| < 0.2	11,956.59 ± 1,275.73	12,067.59 ± 1,159.32	0.38
	0.2 ≤ |iLI| < 0.4	7,114.88 ± 311.36	7,292.59 ± 166.80	0.07
	0.4 ≤ |iLI| < 0.8	7,527.00 ± 571.83	7,860.35 ± 772.45	0.03
	0.8 ≤ |iLI|	5,105.24 ± 630.03	4,594.53 ± 1,046.44	0.096

*NLL, number of the left-lateralized laterality index; NRL, number of the right-lateralized laterality index; iLI, intrinsic laterality index*.

**Table 4:** Statistical analysis of the mean value of the iLI at different threshold values using the paired-samples *t*-test.

	Groups	MLL	MRL	*P*-value
Healthy control	0 < |iLI| < 0.2	0.0931 ± 0.0018	0.0933 ± 0.0017	0.42
	0.2 ≤ |iLI| < 0.4	0.2913 ± 0.0013	0.2916 ± 0.0012	0.24
	0.4 ≤ |iLI| < 0.8	0.5783 ± 0.0025	0.5794 ± 0.0026	0.10
	0.8 ≤ |iLI|	1.0684 ± 0.0202	1.0496 ± 0.0249	0.02
Mild cognitive impairment	0 < |iLI| < 0.2	0.0938 ± 0.0016	0.0938 ± 0.0017	0.95
	0.2 ≤ |iLI| < 0.4	0.2917 ± 0.0014	0.2921 ± 0.0013	0.07
	0.4 ≤ |iLI| < 0.8	0.5779 ± 0.0024	0.5790 ± 0.0025	0.03
	0.8 ≤ |iLI|	1.0737 ± 0.0178	1.0559 ± 0.0218	0.002
Alzheimer’s disease	0 < |iLI| < 0.2	0.0926 ± 0.0018	0.0932 ± 0.0020	0.03
	0.2 ≤ |iLI| < 0.4	0.2913 ± 0.0013	0.2911 ± 0.0015	0.55
	0.4 ≤ |iLI| < 0.8	0.5774 ± 0.0039	0.5781 ± 0.0042	0.39
	0.8 ≤ |iLI|	1.0807 ± 0.0258	1.0538 ± 0.0288	0.004

*MLL, mean value for the left-lateralized laterality index; MRL, mean value for the right-lateralized laterality index; iLI, intrinsic laterality index*.

### Brain Lateralization in the HCs

In the HCs, significant brain functional lateralization was observed between the hemispheres regarding the sum, number, and mean values of the iLI in the 0.8 ≤ |iLI| threshold (*P* = 0.005, 0.009, and 0.02, respectively), which were shifted leftward, and no lateralization was observed using the other three thresholds.

### Brain Lateralization in the Patients with MCI

The leftward asymmetry patterns observed in the HCs using the 0.8 ≤ |iLI| threshold were maintained in the patients with MCI. However, the brain lateralization pattern in the patients with MCI changed when other thresholds were used. Compared with the HCs, the patients with MCI showed a distinct rightward interhemispheric asymmetry using the 0.2 ≤ |iLI| < 0.4 and 0.4 ≤ |iLI| < 0.8 thresholds, with *P*-values equal to 0.03 and 0.01 for the sum of iLI and 0.04 and 0.01 for the number. Regarding the mean value of the iLI, the patients with MCI also exhibited a rightward dominance using the 0.4 ≤ |iLI| < 0.8 threshold.

### Brain Lateralization in the Patients with AD

Compared with the HCs, the sum and number of the iLI that showed a leftward predominance in the HCs disappeared (*P* = 0.06 and 0.096) in the patients with AD. Thus, the normal brain lateralization pattern was lost in the patients with AD. Moreover, an abnormal rightward laterality in the sum and number of the iLI was displayed using the 0.4 ≤ |iLI| < 0.8 threshold (*P* = 0.03 and 0.03), and a rightward dominance in the mean value of the iLI was also observed using the 0 < |iLI| < 0.2 threshold.

In a comparison of the brain functional lateralization in the patients with AD with that in the patients with MCI, an exclusive rightward laterality was observed in the patients with MCI in both the sum and number of the iLI using the 0.2 ≤ |iLI| < 0.4 threshold, and the leftward asymmetry that was observed in the patients with MCI using the 0.8 ≤ |iLI| threshold vanished in the patients with AD in both the sum and number of the iLI.

### Gender Difference of Brain Lateralization

The results showed no statistically significant difference in brain lateralization according to gender (*P* = 0.51) in the HCs. Differences in brain lateralization between males and females in the MCI and AD groups were significant (both *P* = 0.000). The results are illustrated in the Presentation S1 in Supplementary Material.

One-way ANOVA for male and female subjects to examine differences in the iLI among the groups indicated that in both male and female subjects, the difference among the groups was significant (both *P* = 0.000; Presentation S1 in Supplementary Material).

## Discussion

We compared the functional lateralization over the whole brain in patients and controls to identify the distinct pathological changes that occur in patients with MCI and AD. Our study compared the iLI of three groups and investigated the sum, number and mean values of the iLI in 256 pairs of seed regions based on spontaneous brain activity. We observed differences in the patterns of brain lateralization among the HCs, patients with MCI and patients with AD. Moreover, according to the paired-samples *t*-test, the alterations in brain lateralization between the patients with MCI and the HCs were different than the alterations observed between the patients with AD and the HCs. These findings provide new insights that may improve our understanding of the two types of neurodegenerative diseases.

In this study, hemispheric lateralization was calculated according to the iLI, which was significantly different among the three groups. A previous study revealed that the whole-brain lateralization in healthy elderly subjects had a leftward predominance. Figure 4 reveals that the peaks of iLI among the three groups are all on the right side of 0, indicating a leftward tendency. The pattern of brain functional lateralization in healthy elderly subjects may be related to language dominance. The specialization of the left hemisphere in language was one of the earliest observations of brain asymmetry (27). The planum temporale, which is an extension of Wernicke’s posterior receptive language area, exhibits a marked leftward volume asymmetry; the left planum temporale is up to 10 times larger than its right hemisphere counterpart in humans. This form of asymmetry is perhaps the most prominent and functionally significant form of human brain asymmetry (28). In addition, the leftward specialization in language is related to the degree of right-handedness, and approximately 97% of right-handed individuals show left-hemisphere speech and language localization (29). All participants in this study were right-handed and showed leftward lateralization in the most left-lateralized threshold, indicating that this asymmetry may be related to the leftward dominance of language.

A decrease in the hemispheric asymmetry during disease progression has consistently been reported in studies using electroencephalography, near-infrared spectroscopy, and MRI (30–33). This observation was also confirmed in this study. In the present study, the normal leftward lateralization observed in the most left-lateralized threshold (|iLI| ≥ 0.8) in healthy elderly subjects and patients with MCI disappeared in the patients with AD likely due to the disease-induced damage in the left hemispheric cortex. The left hemisphere has been reported to be more susceptible to neurodegenerative diseases than the right hemisphere (34–37). In patients with AD, the gray matter loss initially emerges in the entorhinal and temporal–parietal cortices and progresses into the frontal and sensorimotor territories as the disease progresses. This sequence occurs in both hemispheres, but regions in the left hemisphere are affected earlier and more severely. The loss of gray matter in the left hemisphere occurs more rapidly than the loss in the right hemisphere (0.79% per year faster) (34). In patients with MCI, the atrophy in the gray matter in the hippocampus, parahippocampal gyrus, and entorhinal cortex was more severe in the left hemisphere than that in the right hemisphere. This result was also consistent with the findings of our previous study, which showed an asymmetric loss of gray matter in the left medial temporal lobe during the progression of MCI to AD, and the loss of gray matter in the left hemisphere was much more severe (35–37).

The disappearance of the normal leftward lateralization in the patients with AD may serve as a biomarker of the substantial loss of left-lateralized nerve cells. MCI is the preclinical stage of AD, and different AD stages may have different neural mechanisms. Patients with AD may present with the typical brain changes and symptoms that are manifested in patients with AD, but patients with MCI may show slight changes and symptoms compared to patients with AD. The loss of gray matter in patients with MCI might primarily occur in the left medial temporal lobe rather than in most of the left hemisphere; therefore, the left-lateralized pattern still exists.

We also observed some abnormal right-lateralized thresholds in the patients with MCI and AD. The rightward lateralization in the patients with MCI and AD may be reflected as (1) a relative increase in brain activation within the right hemisphere or (2) a relative decrease in brain activation within the left hemisphere. Besides the differences we observed among the groups using the one-way ANOVA, a trend of increased brain activation within the right hemisphere was observed in the patients with MCI compared with healthy elderly subjects as shown in Tables 2 and 3.

Mild cognitive impairment is considered a transitional stage between healthy aging and early AD. MCI is a stage of progressive global cognitive decline, including the loss of memory, reasoning, and language. Abnormalities in functional integrity and functional compensation coexist in patients with MCI. The right-lateralized characteristics observed in patients with MCI may primarily result from an attempt to compensate, indicating a relative increase in brain activation within the right hemisphere. Increased activity or functional connectivity within the right hemisphere has been observed in patients with MCI at resting state or during various cognitive tasks (38–41). In attention-demanding tasks, patients with MCI exhibit a greater activation in the bilateral posterior parietal and dorsolateral prefrontal cortices than healthy elderly subjects, but the degree to which the activation was increased was greater in the right hemisphere than that in the left hemisphere. In a word memory task, patients with MCI showed a significant increase in the activation of many compensatory regions than HCs; most regions were located in the right hemisphere (38–40). According to a study by Liang et al., patients with MCI may use additional neural resources in the right prefrontal regions to compensate for the losses in cognitive functions (42). This increased activation in the right hemisphere was consistent with the results of our present study to a certain extent because we observed an abnormal right-lateralized pattern in the patients with MCI. Although compensation for brain function was observed in both hemispheres in the patients with MCI in our study, the right hemisphere was likely dominant over the left hemisphere.

The reason for the abnormal right-lateralized pattern might be more complex in patients with AD that in patients with MCI. Compared with the HCs, the mean values of both the sum and number of the left-lateralized or right-lateralized iLI were decreased in the patients with AD using the 0.4 ≤ |iLI| < 0.8 threshold. These decreases may be due to the interaction among the atrophy in the cortex in the left hemisphere (35, 36), the compensation for global cognitive function, and the inability to compensate (39, 43, 44). First, the extensive loss of left-lateralized nerve cells that results in atrophy in the cortex in the left hemisphere may decrease brain activation within the left hemisphere and trigger a relative increase in brain activation within the right hemisphere. Moreover, in semantic and episodic tasks, patients with AD have been shown to recruit bilateral prefrontal cortices rather than the left ventrolateral prefrontal cortex, which is recruited in HCs, and the recruitment of the bilateral prefrontal regions may reflect a more general adaptation to the loss of cognitive resources (45–48). This type of compensation may also enhance right hemispheric activation. In addition, compensation breakdown has been observed in patients with AD (39, 43, 44). Based on the results of our present study, the normal leftward lateralization pattern observed in the HCs and patients with MCI disappeared in the patients with AD; the reduced asymmetry impeded compensation and the interaction between the hemispheres, impairing the working efficiency of the brain (49). Based on the discussion presented above, this impairment may affect brain lateralization. The primary cause of the observed right-lateralized alterations in the patients with AD remains unclear and provides motivation for our further studies.

Previous studies revealed that gender differences may influence brain asymmetry; however, the results were controversial. Herron and colleagues found males had a larger amygdala, with subtle differences in some cortical regions as well (50). In another study, Luders et al. considered regional hemispheric differences to be slightly pronounced in males compared with females in most regions of the cortex; however, statistical tests revealed there was no significant interaction between gender and hemisphere (51). Another study examined the asymmetry of hippocampal volume as well as other temporal lobe structures in 194 subjects and found similar results (52). Functional MRI studies have revealed the presence of slight sex differences in intrinsic activity asymmetry of the human brain (21). In our study, HCs showed no sex differences, whereas patients with AD and MCI showed significant sex differences. We consider this result to be partly influenced by the sex ratio in our sample. The male to female ratio was close to 1:1 in HCs, while females accounted for a larger proportion of the patients with MCI and AD. In both the male and female subjects, there were differences among the groups. Therefore, the lack of differences in the sum, number and mean values of the iLI among the groups may partly have been influenced by the sex ratio.

Our study had several limitations. First, we chose MRI data from ADNI form same MRI machine with same parameter, and that is why sample size was small. Second, our study mainly focused on brain functional lateralization. Some fMRI investigations have recently suggested that functional results are potentially influenced by structural differences between groups (53, 54) because the structural pattern differences between the two hemispheres are not symmetric (27). Unfortunately, the relationship between brain structural asymmetry and brain lateralization, which is our primary objective, has not been identified to date. Future studies that combine brain lateralization with structural asymmetry techniques will be helpful for exploring the relationship between abnormal brain lateralization and the abnormalities in brain structural asymmetry in patients with MCI and AD. Third, our study only showed a shift in the pattern of brain lateralization over the whole brain of patients with MCI and AD; we did not identify primary regions related to this type of change. We should investigate the abnormal brain lateralization in certain brain regions to precisely illustrate the complex functional deficits in patients with MCI and AD. In the future, we will mainly study aberrant brain lateralization in certain susceptible regions (e.g., the hippocampal, entorhinal, and temporal–parietal cortices) using the same method. Finally, in the current study, the global signal was used as a regressor to remove the associated variance from the fMRI analyses (49). However, much debate exists regarding regressing the global mean signal. Further studies are needed to resolve this debate. In addition, all participants in this study were right-handed, and researchers have not determined whether the same alterations in brain lateralization occur in left-handed subjects. We may examine this issue in our future studies. In addition, because our study has a small sample size, larger samples should be investigated in the future.

In summary, to the best of our knowledge, this is the first study to quantify functional laterality based on fluctuations in intrinsic activity over the whole brain using fMRI. We observed significant brain lateralization patterns among the groups, and the alterations in brain lateralization that were different between the patients with MCI and the healthy elderly subjects were different from those observed between the patients with AD and the healthy elderly subjects. Brain lateralization was observed in the healthy elderly subjects, who exhibited a leftward predominance. In the patients with MCI, the normal leftward lateralization observed in the HCs still existed, but some abnormal right-lateralized patterns appeared. In patients with AD, the normal left lateralization pattern disappeared, and some abnormal right-lateralized patterns were observed. Our study provides new insights that may improve our understanding of the pathophysiological changes that occur during these two states of the neurodegenerative disease.

## Ethics Statement

We confirm that we have read the Journal’s position on issues involved in ethical publication and affirm that this work is consistent with those guidelines.

## Author Contributions

HL and LZ: manuscript drafting, revision, and editing. QX and XZ: study idea and study design. WM, QL, and XW: data analysis. FW: data acquisition. HL, LZ, and QX contributed equally to this work.

## Conflict of Interest Statement

The authors declare that the research was conducted in the absence of any commercial or financial relationships that could be construed as a potential conflict of interest.

## Footnotes

^1^http://adni.loni.usc.edu.

^2^http://rfmri.org/DPARSF.

^3^http://www.fil.ion.ucl.ac.uk/spm/.

^4^http://restfmri.net
