# Supplementary material for: Changes in Brain Lateralization in Patients with Mild Cognitive Impairment and Alzheimer’s Disease: A Resting-State Functional Magnetic Resonance Study from Alzheimer’s Disease Neuroimaging Initiative
Source: Front Neurol. 2018 Feb 8;9:3. doi: 10.3389/fneur.2018.00003 (PMC5810419; doi:10.3389/fneur.2018.00003)
Supplement: Supplementary file 1 [file Presentation_1.PDF]

Supplementary materials

We compared the iLI according to gender in three groups. Two-sample test was used to evaluate iLI of male and female subjects in each three groups. One-way ANOVA was employed to estimate iLI of the same gender among three groups. HCs showed no statistically significant brain lateralization between male and females subjects (P=0.51). Brain lateralization between male and female in MCI and AD groups was significant (both P<0.00).

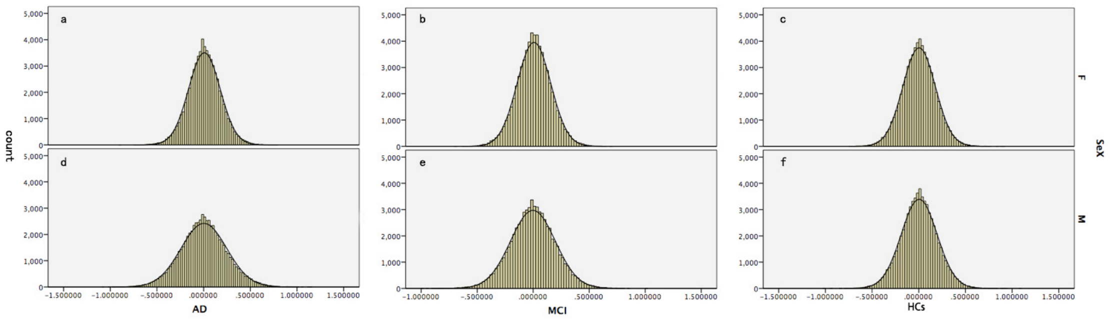

Fig.S1 Sex differences of the iLI distribution. Horizontal axis presents iLI value, vertical axis presents count.
